# Deep Learning Applied to Phenotyping of Biomass in Forages with UAV-Based RGB Imagery

**DOI:** 10.3390/s20174802

**Published:** 2020-08-26

**Authors:** Wellington Castro, José Marcato Junior, Caio Polidoro, Lucas Prado Osco, Wesley Gonçalves, Lucas Rodrigues, Mateus Santos, Liana Jank, Sanzio Barrios, Cacilda Valle, Rosangela Simeão, Camilo Carromeu, Eloise Silveira, Lúcio André de Castro Jorge, Edson Matsubara

**Affiliations:** 1 Faculty of Computer Science, Federal University of Mato Grosso do Sul, Campo Grande 79070900, MS, Brazil; wellingtonvcastro@gmail.com (W.C.); caio.polidoro@aluno.ufms.br (C.P.); lucas.rodrigues@ifms.edu.br (L.R.); edsontm@facom.ufms.br (E.M.); 2 Faculty of Engineering, Architecture and Urbanism and Geography, Federal University of Mato Grosso do Sul, Campo Grande 79070900, MS, Brazil; wesley.goncalves@ufms.br (W.G.); eloiseambiental@gmail.com (E.S.); 3 Faculty of Engineering, Architecture and Urbanism, University of Western São Paulo, Presidente Prudente 19067175, SP, Brazil; lucasosco@unoeste.br; 4 Embrapa Beef Cattle, Brazilian Agricultural Research Corporation, Campo Grande 79106550, MS, Brazil; mateus.santos@embrapa.br (M.S.); liana.jank@embrapa.br (L.J.); sanzio.barrios@embrapa.br (S.B.); cbdovalle@gmail.com (C.V.); rosangela.simeao@embrapa.br (R.S.); camilo.carromeu@embrapa.br (C.C.); 5 Embrapa Instrumentation, São Carlos 13560970, SP, Brazil; lucio.jorge@embrapa.br

**Keywords:** Convolutional Neural Network, biomass yield, data augmentation, phenotyping

## Abstract

Monitoring biomass of forages in experimental plots and livestock farms is a time-consuming, expensive, and biased task. Thus, non-destructive, accurate, precise, and quick phenotyping strategies for biomass yield are needed. To promote high-throughput phenotyping in forages, we propose and evaluate the use of deep learning-based methods and UAV (Unmanned Aerial Vehicle)-based RGB images to estimate the value of biomass yield by different genotypes of the forage grass species *Panicum maximum* Jacq. Experiments were conducted in the Brazilian Cerrado with 110 genotypes with three replications, totaling 330 plots. Two regression models based on Convolutional Neural Networks (CNNs) named AlexNet and ResNet18 were evaluated, and compared to VGGNet—adopted in previous work in the same thematic for other grass species. The predictions returned by the models reached a correlation of 0.88 and a mean absolute error of 12.98% using AlexNet considering pre-training and data augmentation. This proposal may contribute to forage biomass estimation in breeding populations and livestock areas, as well as to reduce the labor in the field.

## 1. Introduction

Monitoring crop parameters like nutrient content, biomass, and plant height is essential for yield prediction and management optimization [1]. In situ measurements of these parameters can be a time-consuming, expensive, and biased task. To assist in plant breeding programs [2] as well as in precision agriculture practices [3], remote sensing technologies have been used in multiple approaches [4,5,6], and, lately, this has expanded with the implementation of UAV (Unmanned Aerial Vehicle) based-data. In recent years, UAV-based images, in conjunction with robust and intelligent data processing methods, are being used as an alternative to the human-visual inspection of agricultural landscapes [7].

The use of machine learning methods [8], such as RF (Random Forest) and SVM (Support Vector Machine) in remote sensing applications has been increasing in the last few years, even in precision farming approaches [9,10,11]. Deep learning is a type of machine learning technique that automatically extracts features, and uses a deeper neural network with hierarchical data representation [12]. This technique uses different structures to perform multiple tasks, and, recently, Convolutional Neural Networks (CNNs) [13] are considered as one of the most important architectures to image and pattern recognition, especially regarding remote sensing related tasks [14,15].

Deep learning was applied in diverse scenarios with crops such as citrus-trees [11,16], canola [17], rice [18], corn [19], bean and spinach [20], and others. There is still a lack of deep learning-based applications in forage crops, specifically using remote sensing data. Although many deep neural networks were proposed to object detection and segmentation in different agriculture practices, few studies implemented this evaluation for biomass estimate for pasture fields. Biomass yield is the main trait evaluated in forage breeding programs, and selection gains can be increased by strategies that allow high-throughput phenotyping in larger breeding populations. In livestock farms, accurately measuring biomass is relevant since the profitability can be strongly affected by it [21].

In agriculture-related problems, Osco et al. [4] estimated nitrogen content in citrus-trees using multispectral UAV-based imagery. A study [22] was able to model water-stress in a vineyard using hyperspectral UAV-based imaging. Another approach [9] proposed a new technique with UAV-based hyperspectral imagery to detect citrus canker disease. Regarding plant detection, a study [23] proposed a method to identify tobacco plants in UAV-RGB-based images automatically. Most of these practices were beneficiated from the usage of machine learning [4,9,22] and deep learning [23] methods combined with UAV data, mainly because of the relatively low cost of the equipment and a high capacity to map plantation fields with very high spatial-resolutions.

Regarding biomass estimation, Bendig et al. [1] proposed an approach adopting CSMs (Crop Surface Models), which are estimated based on the difference of the DSM (Digital Surface Model) and the DTM (Digital Terrain Model), both obtained with UAV Photogrammetry. Going forward, Bendig et al. [24] compared CSMs and VIs (Vegetation Indices) approaches to determine biomass. The authors used three visible band (RGB) VIs: the green red vegetation index (GRVI), the modified GRVI (MGRVI), and the red-green-blue VI (RGBVI). They concluded that GRVI and RGBVI are promising as data input, but the CSMs approach based on plant height returned more accurate results. Ballesteros et al. [25] used canopy cover, crop height, and canopy volume to estimate onion biomass. Batistoti et al. [26] also investigated CSM models to estimate biomass in pasture species, achieving satisfactory results.

Strategies based on plant height are limited because they require DTMs, which are 3D models of the terrain (i.e., without the plants covering the surface). The generation of a DTM can be performed using a GNSS (Global Navigation Satellite System)-based survey [26], which is time-consuming, especially in large areas, or flight-based approaches before initializing the planting process [1]. Although it is not possible to provide more accurate results compared to the CSMs approach, RGB VI-based approaches already demonstrated promising results, and Bendig et al. [24] pointed out that further investigation in this matter is necessary.

Nasi et al. [27] investigated the use of RGB and hyperspectral UAV-based data to estimate biomass. They used the machine learning method RF (Random Forest) combining 3D information, RGB, and hyperspectral imageries. The authors achieved the best performance by integrating hyperspectral and 3D features. However, they highlighted that the integration of RGB images and the 3D features also provided excellent results. Li et al. [28] conducted experiments using the RF learner by joining 3D information, RGB, and hyperspectral data for estimating potato plantations biomass. Unfortunately, hyperspectral sensors are expensive, and, in a general sense, they are not available for a wide range of applications.

Previous researches related to our proposal used conventional machine learning methods, which require handcraft features [29]. Although some of them returned good accuracies, there is still a need for more robust models to be proposed in this task. Based on a review analysis, Kamilaris and Prenafeta-Boldú [29] verified that deep learning methods outperformed traditional machine learning in several agriculture applications. In this sense, the estimation of biomass using deep learning is still scarce in the literature. In previous work, Ma et al. [30] assessed a deep learning architecture based on VGGNet to predict the above-ground biomass of winter wheat. The authors used an RGB camera on a terrestrial tripod, which somewhat limits the application in larger areas. Nevertheless, this experiment demonstrated the potential of CNN to ascertain this task and stated that the proposal of novel methods on this theme is still necessary.

To the best of our knowledge, no literature focused on investigating deep learning-based biomass estimation methods using UAV-RGB orthoimages in tropical forages. Approaches using RGB orthoimages seem to be an interesting practice since they have higher spatial resolution compared to images from other types of sensors, and it does not rely on the 3D information of the canopy, reducing the amount of data necessary to perform the said task. The contribution of this study is to propose a deep learning approach to estimate biomass in forage breeding programs and pasture fields using only UAV-RGB imagery and AlexNet and ResNet deep learning architectures. We also compared the results with VGGNet, used in previous work [30] on biomass estimation. The rest of the paper is organized as follows. Section 2 presents both the materials and methods implemented in this study. Section 3 presents and discusses the results obtained in the experimental analysis. Finally, Section 4 summarizes the main conclusions of our approach.

## 2. Materials and Methods

### 2.1. Study Area and Dataset

**Figure 1:**
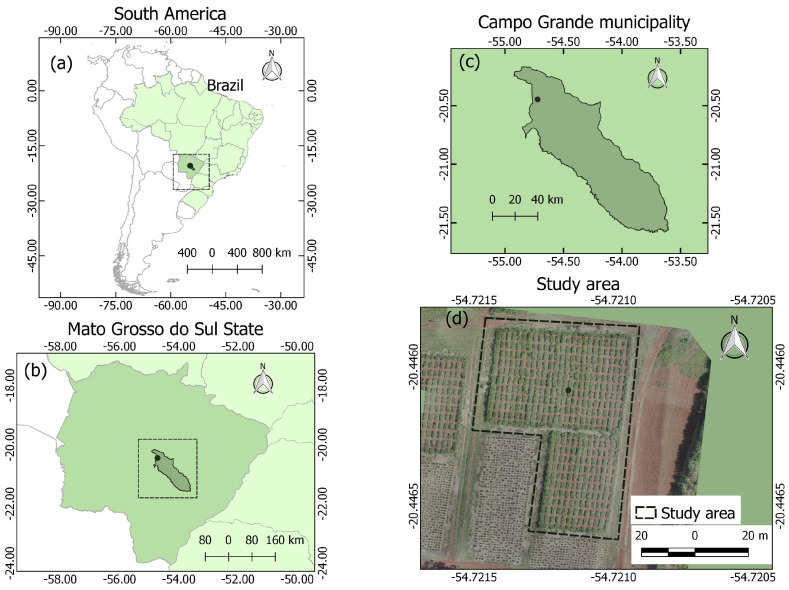
Dataset location considering WGS-84 reference system: (**a**) South America/Brazil; (**b**) Mato Grosso do Sul State; (**c**) Campo Grande municipality and; (**d**) Study area.

**Figure 2:**
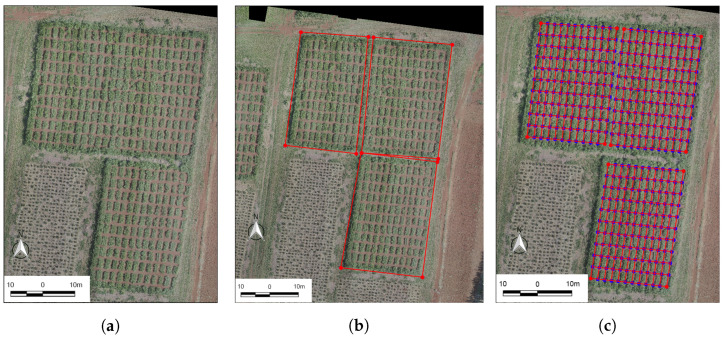
Plots identification procedure: (**a**) Orthomosaic; (**b**) user defined experiment field in red and; (**c**) plots defined using our Python script.

The dataset was formed by images obtained by the UAV DJI Phantom 4 PRO embedded with an RGB digital camera with an image resolution of 5472 × 3648. The area of the experiment is located in Brazilian Cerrado at the Experimental Station of Embrapa Beef Cattle, Campo Grande, Mato Grosso do Sul, Brazil (Figure 1—latitude 20${}^{\circ }$26${}^{\prime }$46${}^{\prime \prime }$ S, longitude 54${}^{\circ }$43${}^{\prime }$16${}^{\prime \prime }$ W) and altitude 535 m. The flight was carried out on 23 January 2019, at around 9 a.m. with a relative height of 18 m, resolving 0.5 cm/pixel. The photos were taken with a frontal overlap of 81% and a lateral overlap of 61%. The orthoimage (Figure 2b) was generated using the Pix4D software based on the SfM (structure-from-motion) and MVS (multi-view stereo) techniques.

The experiment was composed of 110 genotypes representing a high genetic diversity of the species *Panicum maximum* (syn. *Megathyrsus maximus*), an important tropical forage grass for livestock production [31]. These genotypes are grouped by 86 full-sib progenies, ten sexual and ten apomictic progenitors along with four commercial cultivars (Mombaça, MG12 Paredão, BRS Quenia, and BRS Tamani). All the genetic material, with exception of MG12 Paredão, was developed by the *P. maximum* Breeding Program of Embrapa. A randomized complete block design was used with three replications, totaling 330 plots. Each plot consisted of two rows of 2.0 m with 0.5 m apart. Each row contained five plants spaced by 0.5 m between plants, totaling ten plants per plot. Plots were 1.0 m apart, representing an area of 4.5 m${}^{2}$. We evaluated total green matter yield trait as ground truth data. Each plot was harvested 0.2 m from the soil, and the green material was weighted in kg·plot${}^{-1}$ using a field dynamometer and converted in kg·ha${}^{-1}$ on 25 January 2019. For term simplicity, this trait will be named later only as biomass yield.

Figure 2 shows the plot’s definition procedure. We developed a python script tool (https://github.com/wvmcastro/tiffviewer) named field plot cropper (fPlotCropper). The inputs of the tool are: the orthomosaic, the number of blocks in the image, the number of lines, and columns within each block, and the user-defined rectangular polygons (Figure 2b). The result can be seen in Figure 2c.

Dealing with orthoimages can prove to be a challenging task from the computational point of view, as images of this type can go beyond gigabytes in size. In this regard, the use of specific software to reduce these images is necessary. For this, fPlotCropper uses Matplotlib [32] packages for viewing the orthoimages and capturing mouse events, which the user produces when defining the four corners of each block. fPlotCropper also uses Rasterio [33] for reading and writing of the images, along with the Python 3 programming language [34].

**Figure 3:**
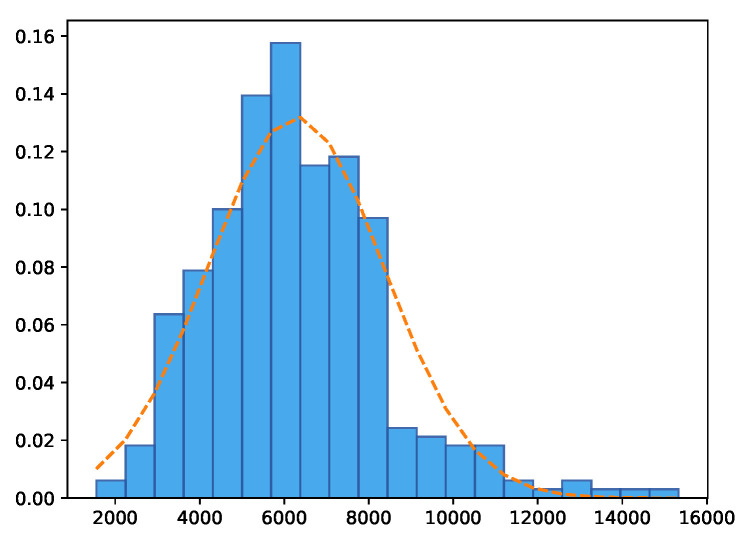
Class attribute *y* distribution-biomass in kg·ha${}^{-1}$.

The presented proposal uses biomass yield as a class attribute *y*. Figure 3 plots the histograms of the *y* data distribution of the 330 plots of the experimental station.

After this pre-processing step, where plots were correctly cropped and identified, we then proceeded with the experimental evaluation described in the next sections.

### 2.2. Deep Learning Approach and Experimental Setup

Conventional machine learning techniques require considerable domain expertise and careful engineering to extract meaningful features to train the models. According to LeCun [35], deep learning methods can learn convolutional filters that can obtain those meaningful features from the images. The advantage is that the learning process incorporates the learning of convolutional filters that replaces the feature extractors designed by human engineers. This approach requires minimal engineering by hand and has achieved a state of the art results in many areas of machine intelligence [35]. Therefore the filters to emphasize and extract essential features are now inside the model, allowing the model to process images in its raw format.

However, the training of CNNs requires large datasets, such as ImageNet, with 14 million images. In the forage literature, to the best of our knowledge, there is no such dataset. In green biomass estimation, the sample size is relatively small for deep learning standards. This limitation relates to obtaining the ground truth of the field biomass, which requires expensive laboring work to harvest and weigh each plot’s biomass.

In our study, we have only 330 plots. Training CNNs with a small sample size is a challenging task. The literature points to fine-tuning approaches using a pre-trained model [36], learn smaller models [37], and perform data augmentation [38]. We address the learning problem by trying these three approaches. We selected AlexNet [39] (8 layers), a popular and relatively small convolutional neural network, and ResNet [40] (18 layers), both with and without a pre-trained model, and with and without data augmentation. We designed the experimental evaluation, not only to show the individual accuracy rate of each model but also to measure how effective is each of these three approaches. As a baseline we use VGGNet  [41] (11 layers), because it was used for the same purpose in other species [30].

We used AlexNet, ResNet18 and VGGNet from PyTorch [42]. The last fully connected layer architectures was changed so that the models were adapted to a regression problem. The settings for each experiment can be seen in Table 1. In all experiments, the Adam [43] optimization method was used, with the descending gradient algorithm, in a fixed learning rate of 0.001, constant $\beta_{1}=0.9$, $\beta_{2}=0.999$ and $\epsilon =10^{-8}$. The number of epochs was defined empirically using early stopping evaluated every 100 epochs. The pre-trained models are PyTorch pre-trained model on ImageNet (see https://pytorch.org/docs/stable/torchvision/models.html), where we loaded these pre-trained weights and fine-tuned these models using the training set. Models without pre-training were fully trained using the training set.

Considering the relatively restricted number of 330 plots (examples), we performed all experiments using ten-fold cross-validation, since cross-validation produces better generalization error than hold-out [44,45]. To pursue more robust models, we trained them using the data augmentation technique. We named *augmented horizontally* (augmented h) for regular data augmentation, where the images were flipped from left to right. We named *augmented horizontally and vertically* (augmented hv) for data augmentation, where the images were also flipped from top to bottom. The models without data augmentation were named original in Table 1.

All models were trained to adopt the MSE (mean square error) as the loss function. The mean square error evaluated values between the actual value of biomass, in kg·ha${}^{-1}$ ($y_{i}$), and the value predicted by the model was used ($\hat{y_{i}}$). The formula is in the Equation (1),

(1) $$\varepsilon =\frac{1}{n}\sum_{i=0}^{n}{\left(y_{i}-\hat{y_{i}}\right)}^{2}$$

where *n* is the number of examples, $y_{i}$ is the true score, $\hat{y_{i}}$ is the predicted score, and $y_{i}\in \left[1556,15,333\right]$, which means that the $y_{i}$ varied from 1556 kg·ha${}^{-1}$ to 15,333 kg·ha${}^{-1}$. We used a desktop with a NVIDIA Titan X GPU (12 GB), Intel i7-6800K 3.4 GHz CPU, and 64 GB of RAM.

We assess the regression problem using MAE (mean absolute error), MAPE (mean absolute percentage error), R (Pearson correlation). However, these metrics can hide predictions biased towards higher or lower values than the true prediction. The same can occur with the graphs in Section 3, where high-density regions can overlap points. This limitation motivates the evaluation of the results under RROC (Receiver Operating Characteristic Curves from Regression) [46]. We also evaluate the results using histograms (Section 3.3). The number of *bins* was determined using the elbow rule [47] from the partitioning of the samples’ real values.

For visual inspection of the model activations, the last convolutional layers of CNNs retain spatial information and high-level semantics of the CNN [48]. Looking at these layers, it is possible to highlight the class-discriminative regions of the images. One of the first methods to emphasize the discriminative areas of the images was the CAM (class activation mapping) [48]. However, CAM works only on CNNs without fully connected layers architectures. One year later, Grad-CAM [49] was proposed, enabling the use of fully connected layers without architectural changes or re-training. We applied Grad-CAM and displayed some results in Section 3.4.

## 3. Experimental Results Evaluation

We divided our evaluation into four parts: (1) evaluation of the results using standard metrics MAE, MAPE, and R, and the graphs of predicted versus real values; (2) a visual representation of the error by ROC Regression curves; (3) the histograms of the predictions; and (4) the heat map of the feature map activation for visual inspection.

### 3.1. Standard Evaluation: MAE, MAPE, R, and Graph of Predicted Versus Real

Table 2 shows the mean and standard deviation of mean absolute error, mean absolute percentage error, and Pearson Correlation of ten-fold cross-validation. The results indicate four groups of outcomes. The best first group represents AlexNet pre-training, Experiment #7, #8, and #9. These results are among the top 3 results showing an average MAE lower than 768.75 kg·ha${}^{-1}$. The second best group is also from AlexNet, but without pre-training, Experiment #1, #2, and #3 with average MAE lower than 924.48. The third and fourth groups are the ResNet18 results, where the MAE is higher than 1000. Overall and surprisingly, the results of AlexNet are better than ResNet18. The VGGNet (baseline) was considered only with pre-training and data augmentation because we verified that this contributed to the improvement of the other models. The AlexNet also outperformed VGGNet11, which presented an average MAE of 825.94 kg·ha${}^{-1}$. Experiment #9 presented the best absolute result.

**Figure 4:**
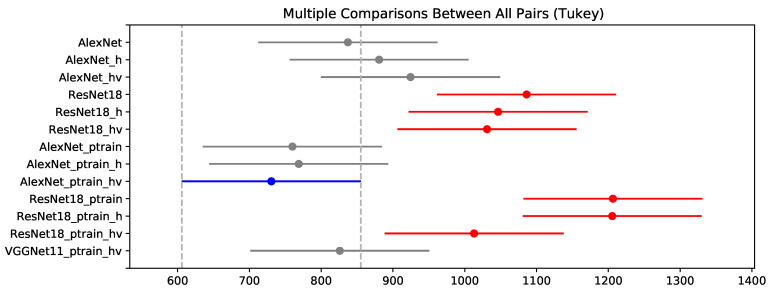
Mean and the 95% confidence interval of a pos-hoc Tukey’s HSD test performed on MAE results.

We then proceed with the experimental evaluation performing a one-way ANOVA test. We obtained an F-statistic of 9.81, and with a *p*-value of 5.16 × $10^{-13}$ < 0.05, we can reject the null hypothesis that the models have are equal performance using MAE. We continue the evaluation with Tukey’s pos-hoc test to find the differences among the experiments. Figure 4 shows the Tukey’s HSD.

The results that differ significantly with AlexNet_ptrain_hv are red, the insignificant difference are gray. Therefore the results indicate that AlexNet_ptrain_hv has a significant difference with all ResNet18 results. The pre-trained models showed a slight improvement in the AlexNet result but not in ResNet. The ResNet results, except for ResNet18_ptrain_hv, the pre-training deteriorated the results. For data augmentation hv, we can see a small improvement in pre-trained models of AlexNet and ResNet. For the AlexNet without pre-training, the use of data augmentation worsens the results.

**Figure 5:**
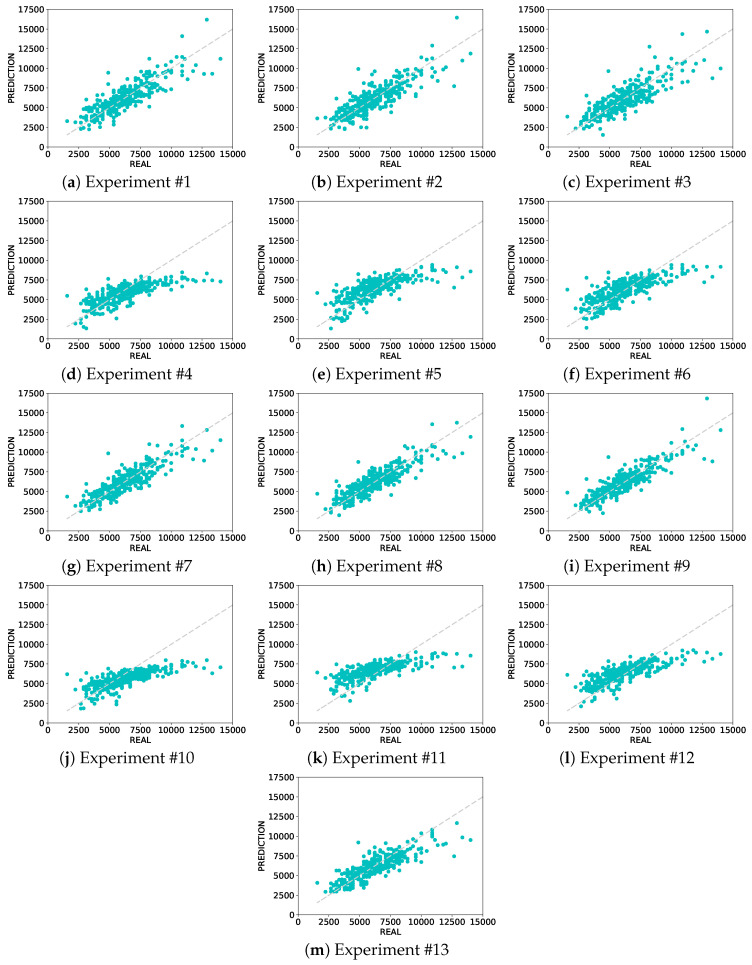
Predicted vs. real plots.

The mean and standard deviations of MAE, MAPE, and R assume Gaussian distributions. Plotting the real values and the prediction in the same graph can be a more precise visualization of the results, such as point cloud density and outliers.The graphs presented in Figure 5 show exactly these visualizations, where points are pair of $\left(y,\hat{y}\right)$. In a situation of perfect predictions, these graphs would be perfect lines (1:1).

In all graphs, the models show more spread results on the top right and a high concentration of points in the center of the diagram, which corroborates with the real class *y* (Figure 3) where the numbers of biomass higher than 11,000 kg·ha${}^{-1}$ are scarce, and the average value of *y* is close to 6000 kg·ha${}^{-1}$.

When comparing the graphs of AlexNet and ResNets, we can see that all ResNet results (Experiments #4, #5, #6, #10, #11, and #12,) show a point cloud lying more to the bottom right, indicating that ResNet has difficulties predicting values higher than 10,000 kg·ha${}^{-1}$ than AlexNet. The pre-trained AlexNet (Experiments #7, #8, and #9 ) shows a narrow corridor of points close to the ascending diagonal (dotted line). The narrowest point cloud and closer to the dotted line seems to be the Experiment #9, which confirms the results of the absolute number of MAE, MAPE, and R.

### 3.2. ROC Regression

**Figure 6:**
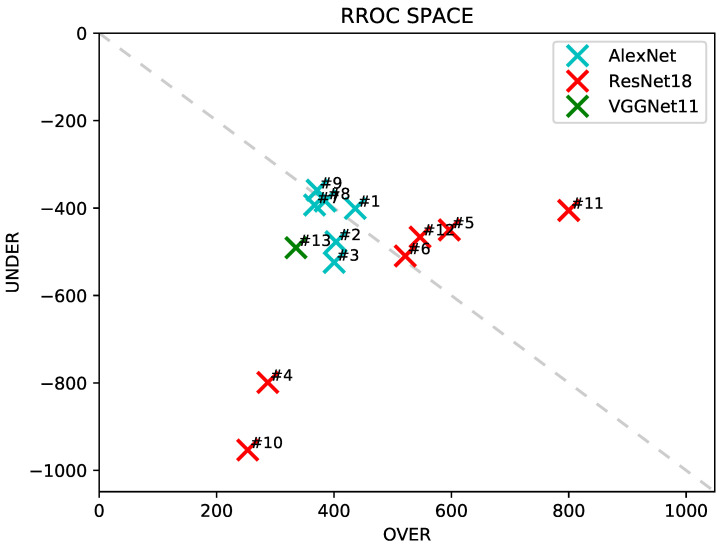
ROC (Receiver Operating Characteristic) for regression. Points closer to (0,0) mean better results.

Figure 6 shows RROC, where the closer to the (0,0) point, called RROC heaven, the better the model. Closer the results to the dotted line UNDER+OVER=0, the less biased the model is to predict values below or above the true regression values.

Among the results with pre-training (Figure 6), AlexNet original, AlexNet augmented h, and AlexNet augmented hv (#7, #8, and #9) are closer to (0,0). ResNet18 pre-trained augmented h (Experiment #11) is above the dotted line, indicating better results for UNDER the prediction than OVER the prediction. In practice, this is equivalent to an average prediction of $\hat{y}$ slightly higher than the true prediction *y* for Experiment #11. This interpretation may be counter-intuitive when we look at Figure 5k due to the points on the middle right of the graph, however looking closely, we can see high-density points in the middle left, that corroborates with the RROC result. The other way around occurs with ResNet18 original (Experiment #4) and VGGNet11(Experiment #13), where the results are below the dotted line.

When comparing AlexNet (light blue) and ResNet (red), we can see that AlexNet points are closer to the (0,0). All ResNet points are further away from the AlexNet points and spread over the graph, showing a barrier of points close to the ascendant diagonal.

### 3.3. Histograms

**Figure 7:**
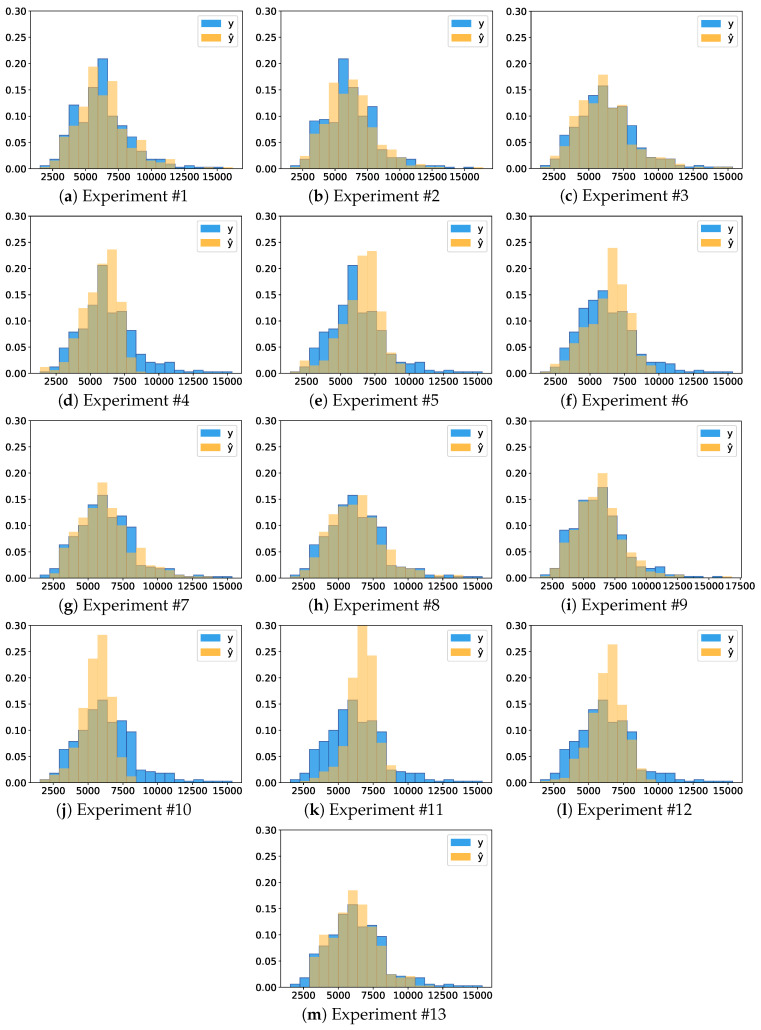
Comparison of prediction $\hat{y}$ vs. real *y* data distribution. Larger intersecting areas between histograms indicates better prediction.

The histogram graphs of Figure 7 show the intersection between the distribution of the real data and the distribution of the predictions of each experiment. The addition of new groups did not significantly increase the representativeness of the data with more than 20 bins, so the number of *bins* was set to 20. The intersection areas between the distributions were calculated for each experiment and are presented in Table 3.

Comparing the ResNet and AlexNet, we can see a peek of orange values on x-axis surrounding the 7500 kg·ha${}^{-1}$ in all ResNet experiments (Experiment #4, #5, #6, #10, #11, and #12). The AlexNet histograms go along the true class (blue) histogram. Experiment #1 and #2 are the only histograms where the highest bin is blue.

Through the Table 3 and Figure 7, it is possible to conclude the superiority of the results of Experiment #9, where the training set used was at least twice as large concerning any other experiment due to the data augmentation.

**Figure 8:**
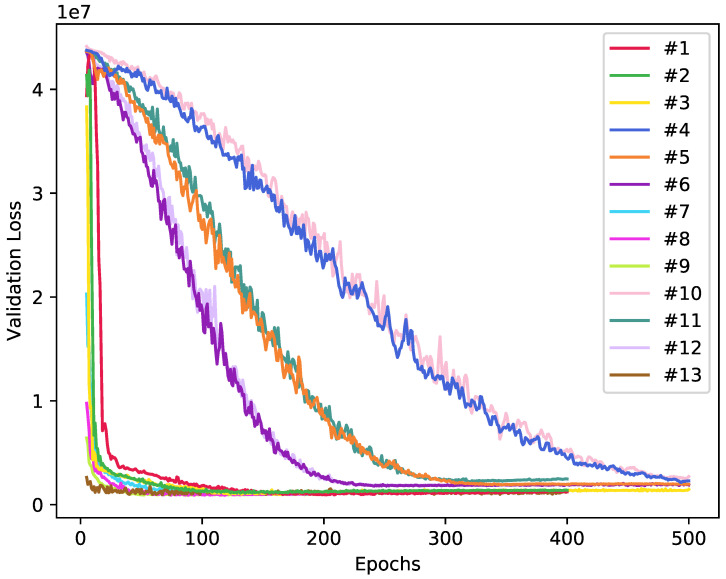
Validation loss over epochs for all experiments.

From the plot of the validation loss given the number of epochs during training presented in Figure 8, it can be observed that Experiments #1, #2, #3, #7, #8, #9, and #13 were the ones to converge the fastest getting to a plateau at around 100 epochs. Experiments #5 and #11 converged at approximately 230 epochs. Experiments #6 and #12 converged after about 300 epochs. Finally, Experiments #4 and #10 were the ones that took the longest to reach the plateau, taking approximately 500 epochs. From this analysis, it is possible to conclude that the AlexNet and VGGNet11 models were able to consistently present better results earlier when compared to experiments using the ResNet18 model.

### 3.4. Visual Inspection

**Figure 9:**
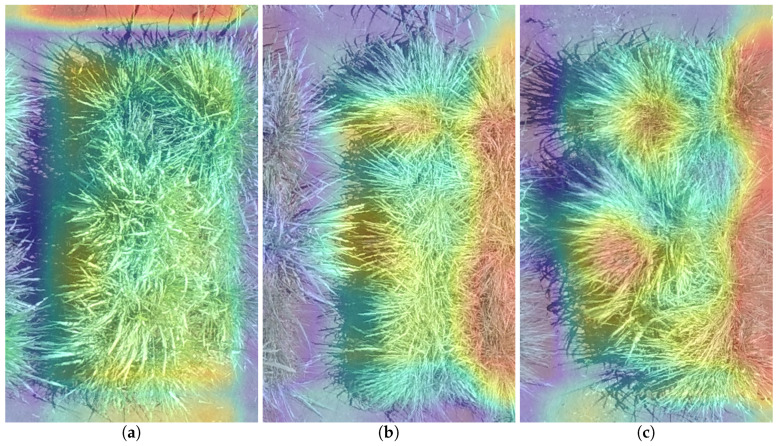
Heatmaps of the top 3 best predictions made by Experiment #9 (Pre-trained AlexNet Model with hv data augmentation): (**a**) First best prediction; (**b**) Second best prediction; (**c**) Third best prediction.

**Figure 10:**
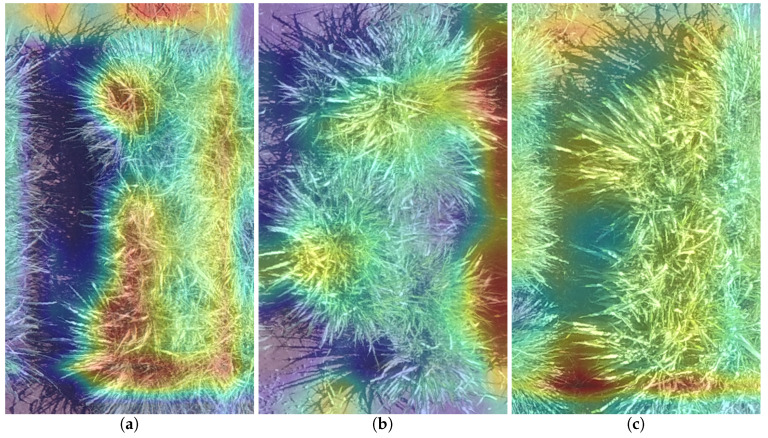
Heatmaps of the top 3 worst predictions made by Experiment #9 (Pre-trained AlexNet Model with hv data augmentation): (**a**) First worst prediction; (**b**) Second worst prediction; (**c**) Third worst prediction.

Figure 9 and Figure 10 show the heatmaps from Grad-CAM on Experiment #9. Warm colors (red) indicate a more class-discriminative region to the prediction, while cold colors (blue) represent the lower class-discriminative region.

Figure 9 shows the three best predictions, where no strong red or blue regions are highlighted. Figure 10 shows the three worst predictions, where extreme values in the neurons, strong red and blues, occur. We believe that these extreme values create a broader range of values that make the regression problem more difficult to solve, worsening the results’ MAE.

### 3.5. Training and Test Time

Finally, we also evaluated the training and test time of one fold of the ten-fold cross-validation procedure. The test set has fix size of 33 examples, and the training set 300, 600, and 900 examples for original, augmented h and augmented hv, respectively. Table 4 shows the results.

The training time needs to be analyzed with the respective number of training steps. It is interesting to see that although the h and hv have two and three times more examples, their training time was not multiplied by a factor of two and three on ResNet. While for the AlexNet, these factors are consistent. The test time stays between 0.39 and 0.67 for AlexNet and ResNet. The testing time of VGG was the highest among the tested results.

## 4. Discussion

Our study focused on a deep learning-based approach to estimate biomass yield in forage fields. Furthermore, we investigated the impact of the data augmentation and pre-training steps on the estimation results. CNN is a state-of-the-art method to evaluate imagery data. We also considered different genotypes of a forage species, which contribute to the heterogeneity of our dataset. For assessing the experiments, we presented regression analysis results with a high correlation between predicted and measured yields (Table 2 and Figure 4), an RROC space comparing the deep networks implemented (Figure 5), an intersection analysis between each experiment (Table 3 and Figure 6) and qualitative information such as heat maps illustrating the best and worst predictions of our data (Figure 8 and Figure 9).

When comparing against other methods to estimate biomass, our approach differentiates from a methodological point-of-view. Up to the point, most approaches considered shallow learners (i.e., conventional machine learning methods) and RGB imagery combined with DSM and DTM models, LiDAR or even vegetation spectral indices from multi and hyperspectral data [1,24,25,26,27,28,50]. As an advantage of adopting deep neural networks is the oversimplification of remote sensing data variety necessary to conduct this estimation. Although the production of input data such as DTM, DSM, spectral indices, and multiple bands is a relatively easy task in remote sensing, the costs to obtain such products, specifically LiDAR and hyperspectral data, are highly-priced when comparing against RGB only imagery. Our deep networks, trained with RGB inputs, can perform similarly or even better than most traditional methods and data previously described. This is an important advance for agricultural remote sensing approaches. The caveat of the proposal and other CNN-based approaches are the requirements of computational intensive procedures, often requiring the use of GPUs.

Compared to the previous study that used 3D information to estimate the biomass for the same species [26], we achieved more accurate results. Batistoti et al. [26] achieved an R2 of 0.74 considering a limited number of plots (four in total). Another study [51] estimated yield with proximal sensing equipment in a heterogeneous sward structure of grasslands and applied a MLPSR (Multiple Partial Least Square Regression) approach, which returned an R2 of 0.69. In estimating biomass from legume-grass, Wachendorf et al. [52] produced high accuracies with a similar approach as Moeckel et al. [51], returning accuracies up to 0.95 for specific models. Another study [53] considered machine learning techniques to estimate grassland biomass in spectral data, with an RMSE equal to 71.2 t·ha${}^{-1}$. This indicates that our UAV-based RGB dataset, in conjunction with more robust methods (i.e., deep neural networks), can be compared against expensive measurement remote systems, considering even terrestrial measurements.

In this study, we implemented two convolutional networks (AlexNet and ResNet18) and tested as a baseline another network (VGGNet11), as stated in the previous sections. To evaluate the impact of samples in our approach we tested whether pre-trained models and data augmentation produced significant improvements in its accuracy. Our results indicated that the AlexNet method performed better. A possible explanation for this is that the ResNet18 method, although being a deeper network than the implemented AlexNet, was unable to represent the pre-trained problem with its convolutional filters properly. In other words, it was not able to modify its layers with enough precision. Results without pre-training steps were also not sufficient. This demonstrates how the lack of data for training impacted its performance. Nevertheless, the evaluation of different pre-processing steps (with and without data augmentation and pre-training) resulted in essential implications for integrating agronomic measurements collected in the field with these robust methods in remote sensing RGB imagery.

The VGGNet11 performance was calculated to be compared against the accuracy obtained in a previous paper [30]. In this paper [30], more traditional approaches were also compared against this deep learning method. These approaches were related to spectral vegetation indices and 3D models as standalone data to estimate biomass yield. Even so, it was demonstrated that the VGGNet11 outperformed these traditional approaches. Here, our proposal focused mostly on evaluations with the AlexNet and ResNet18 networks throughout the experiment. We noticed that in both methods, data augmentation improved the overall performance in estimating biomass yield. As a result of this outcome, we also implemented data augmentation in the VGGNet11 network. Nonetheless, our analysis (Figure 5) demonstrated that the AlexNet method was superior to the deep learning method implemented in [30], even with data augmentation. This may be an indicator that this type of approach with RGB imagery performs better with shallow architectures.

It is possible to observe that the models used were able to return a high correlation between the RGB images of the plots and the real yield value (Table 2 and Figure 4). This study is the first approximation of its kind. We believe that many aspects to be evaluated in future research. Although we stated the importance of RGB data in an economic point-of-view, we do not disregard the impact of other types of remote sensing methods to increase the accuracy of deep learning-based neural networks. The use of 3D reconstruction data from point-clouds can also be explored in the future since biomass has a strong relationship with the volume of the plant. This data insertion could assess whether there are performance gains in the prediction and infer the density of the plant, a significant characteristic for researchers in the area. Regardless, the results utilizing only RGB and the cross-validation method indicate the capability of the proposed method in this approach.

## 5. Conclusions

Until this moment, this paper’s proposed approach is the first research that implemented and evaluated a CNN-based architecture, combined with high-resolution UAV RGB images, for the prediction of biomass yield considering different forage genotypes.

Two regression models based on CNNs (Convolutional Neural Networks) named AlexNet and ResNet18 were evaluated, and compared to VGGNet—adopted in previous work in the same thematic for other grass species. The predictions returned by the models reached a correlation of 0.88 and a mean absolute error of 12.98% using AlexNet considering pre-training and data augmentation. Comparing the achieved results to a previous study that was based on 3D information to estimate the biomass for the same species [26], we achieved more accurate results.

In conclusion, the models used were able to establish a high correlation between the images and the biomass value measured in the field. This demonstrates how feasible the proposed approach is to predict forage yield at highly detailed RGB imagery, producing accuracy comparable to more expensive approaches with both aerial and proximal remote sensing.

Since this is the first study of its kind, there are many aspects to be evaluated in future research. It is worth noting that the models developed here are not yet ready to be deployed in commercial production. Although cross-validation has been used in all experiments, the dataset is still considered small. However, the results obtained are strong indications of the method’s future success in more significant variations of forage crop datasets. Experiments using datasets from different locations and weather conditions are essential to provide more generalized models, and we intend to conduct this in future work.

## Figures and Tables

**Table 1:** Experimental setup.

#Experiment	Model	Batch Size	Data-Set	Epochs
1	AlexNet	256	original	400
2	AlexNet	256	augmented h	400
3	AlexNet	256	augmented hv	500
4	Resnet18	128	original	500
5	Resnet18	128	augmented h	500
6	Resnet18	128	augmented hv	500
7	AlexNet Pre-Trained	256	original	200
8	AlexNet Pre-Trained	256	augmented h	200
9	AlexNet Pre-Trained	256	augmented hv	200
10	ResNet18 Pre-Trained	128	original	500
11	ResNet18 Pre-Trained	128	augmented h	400
12	ResNet18 Pre-Trained	128	augmented hv	400
13	VGGNet11 Pre-Trained	64	augmented hv	400

**Table 2:** Experimental results. Experiment #9’s results present the lowest MAE (mean absolute error) and highest correlation. It is good to remind the reader that $y_{i}$, ranging from 1556.00 kg·ha${}^{-1}$ to 15,333.00 kg·ha${}^{-1}$, therefore MAE of 730 represents a variation of 730 kg·ha${}^{-1}$ in this range of values.

#Experiment	Model	Mean Absolute Error	Mean Absolute Percentage Error	Correlation (*r*)
1	AlexNet	837 ± 106	14.58 ± 2.52	0.84 ± 0.03
2	AlexNet h	880 ± 202	15.11 ± 3.24	0.83 ± 0.06
3	AlexNet hv	924 ± 143	15.48 ± 2.30	0.82 ± 0.05
4	ResNet18	1086 ± 219	17.70 ± 3.41	0.74 ± 0.06
5	ResNet18 h	1046 ± 107	19.01 ± 2.77	0.74 ± 0.06
6	ResNet18 hv	1031 ± 153	18.76 ± 4.28	0.75 ± 0.06
7	AlexNet Pre-Trained	759 ± 102	13.23 ± 2.23	0.87 ± 0.05
8	AlexNet Pre-Trained h	768 ± 123	13.54 ± 2.88	0.87 ± 0.03
9	AlexNet Pre-Trained hv	730 ± 59	12.98 ± 2.18	0.88 ± 0.04
10	ResNet18 Pre-Trained	1206 ± 233	19.46 ± 5.15	0.73 ± 0.04
11	ResNet18 Pre-Trained h	1205 ± 194	23.16 ± 4.80	0.71 ± 0.07
12	ResNet18 Pre-Trained hv	1012 ± 128	18.58 ± 2.34	0.77 ± 0.05
13	VGGNet11 Pre-Trained	825 ± 152	13.89 ± 3.09	0.84 ± 0.04

**Table 3:** Intersection areas of the histograms shown in Figure 7.

**Experiment**	1	2	3	4	5	6	7	8	9	10	11	12	13
**Intersection Area**	0.83	0.83	0.91	0.78	0.72	0.78	0.89	0.89	0.92	0.68	0.62	0.76	0.90

**Table 4:** Training and test time on one-fold of the cross-validation procedure.

#Experiment	Model	Training Time (min)	Test Time (s)
1	AlexNet	35.8	0.39
2	AlexNet h	95.6	0.46
3	AlexNet hv	122.2	0.40
4	ResNet18	60.2	0.47
5	ResNet18 h	131.3	0.54
6	ResNet18 hv	133.7	0.53
7	AlexNet Pre-Trained	15.8	0.37
8	AlexNet Pre-Trained h	36.2	0.44
9	AlexNet Pre-Trained hv	43.7	0.50
10	ResNet18 Pre-Trained	58.1	0.47
11	ResNet18 Pre-Trained h	102.2	0.67
12	ResNet18 Pre-Trained hv	104.4	0.43
13	VGGNet11 Pre-Trained hv	372.2	0.81
